# Protein *N*-Glycosylation Traits Combined With CA19-9 Accurately Distinguish Pancreatic Cancer Cases From Healthy Controls and Benign Pancreatic Diseases

**DOI:** 10.1097/MPA.0000000000002517

**Published:** 2025-05-23

**Authors:** Aleksander M. Bogdanski, Derk C.F. Klatte, Monique E. van Leerdam, Christa M. Cobbaert, Bart E.P.B. Ballieux, Jeanin E. van Hooft, Kristin E. Clift, Manfred Wuhrer, Wilma E. Mesker, Yan Bi, Michael B. Wallace, Yuri E.M. van der Burgt

**Affiliations:** * Department of Gastroenterology and Hepatology, Leiden University Medical Center, Leiden, The Netherlands; † Department of Gastroenterology and Hepatology, Mayo Clinic, Jacksonville, FL; ‡ Department of Gastrointestinal Oncology, Netherlands Cancer Institute, Amsterdam; § Department of Clinical Chemistry and Laboratory Medicine; ∥ Center for Proteomics and Metabolomics; ¶ Department of Surgery, Leiden University Medical Center, Leiden, The Netherlands

**Keywords:** pancreatic cancer, early detection, biomarker, glycans, CA19-9

## Abstract

**Objectives:**

New methods are needed to detect pancreatic ductal adenocarcinoma (PDAC) earlier to improve outcomes. We previously reported that a panel of protein *N*-glycosylation traits (NGTs) discriminated PDAC from healthy controls with an area under the curve (AUC) of 0.81–0.88. However, it remained unclear whether this panel accurately differentiates PDAC from other benign pancreatic disorders. Our study aims to evaluate the performance of the NGT panel in combination with CA19-9 in a diverse cohort, including PDAC cases, healthy controls, and controls with benign pancreatic disorders.

**Methods:**

Protein *N*-glycosylation profiles were determined in plasma samples using an in-house developed mass spectrometry assay. CA19-9 levels were measured using a routine immunoassay test. Results of total plasma NGTs and CA19-9 were evaluated separately as well as in combination. Logistic regression was performed to calculate odds ratios (ORs), AUC, sensitivity, and specificity to determine the performance of NGTs and CA19-9 in distinguishing PDAC from controls.

**Results:**

In total, 221 individuals were included: 45 (20.4%) with PDAC, and 176 (79.6%) controls (53 healthy and 123 with benign pancreatic disease). The AUC for differentiating PDAC from the total control cohort based on the combination of the NGT panel and CA19-9 was 0.94 (95% CI, 0.90–0.97), with a sensitivity of 0.89 (95% CI, 0.78–0.98) and specificity of 0.86 (95% CI, 0.81–0.91). Comparison of PDAC cases with healthy controls only resulted in an AUC of 0.96 (95% CI, 0.93–0.99), with a sensitivity of 0.84 (95% CI, 0.73–0.93) and specificity of 0.98 (95% CI, 0.94–1.00).

**Conclusions:**

Both plasma NGTs and CA19-9 distinguish PDAC from a diverse control cohort. The accuracy further improves when these readouts are combined, showing promise for future early detection methods.

Pancreatic ductal adenocarcinoma (PDAC) is characterized by the lowest 5-year survival rate of any cancer type, at only 4.2%.^1,2^ This poor prognosis results from an often late diagnosis due to the lack of symptoms in early-stage PDAC.^3,4^ Consequently, diagnosis is often delayed until curative treatment is no longer possible.^3,4^ This emphasizes the need for early detection. However, population-wide screening is not feasible due to the relatively low incidence of PDAC and the lack of accurate biomarkers.^3,5^ Despite this, PDAC ranks as the third leading cause of cancer-related deaths worldwide, and is projected to become the second by 2030.^3^ This underscores the need for novel strategies to address this growing public health concern.

In contrast to the implementation of population-wide screening, surveillance in high-risk individuals (HRIs) is indicated due to the significantly elevated incidence of PDAC in these groups.^6^ Currently, imaging-based surveillance programs for PDAC have been established for certain HRIs and have demonstrated added value in terms of overall survival when compared with no surveillance.^2^ Nevertheless, the ability to differentiate between benign and malignant (pre)cursor lesions based on imaging alone remains a significant challenge.^7^ In fact, the rates for unnecessary surgery in surveillance can reach as high as 9.2%.^6^ In addition, despite surveillance, up to 70% of individuals who develop PDAC are still diagnosed at a late stage (stage II or higher).^8–10^ This shows that surveillance based on longitudinal imaging alone is not sufficient and that additional complementary biomarkers are needed to enable early detection of PDAC.^11^ The most studied blood-based biomarker for PDAC is cancer antigen (CA) 19-9; however, the application is limited to predicting treatment response and monitoring of postoperative progress.^11,12^ The diagnostic performance of CA19-9 alone is suboptimal for early detection or surveillance purposes of PDAC.^11^

Notably, CA19-9 is a glycan epitope that is present on various glycoproteins. Protein glycosylation is a common cotranslational and posttranslational modification that plays a key role in various biological processes since it changes protein structure and function.^13^ Consequently, the analysis of protein glycosylation provides insights into protein function beyond genetic, transcriptomic, and proteomic information.^14^ Altered protein glycosylation may have an influence on or may be caused by tumor growth, differentiation, transformation, adhesion, pathogen recognition, and immune surveillance.^15,16^ Variations in total *N*-glycan levels hold promise in distinguishing individuals with PDAC from those without the disease.^17–19^ More specifically, aberrant glycosylation profiles have been reported on the surface of cancer cells with a potential diagnostic value toward evaluating tumor progression.^20^ In previous studies, we have developed an NGT panel based on the best-performing *N*-glycan–derived features to discriminate between PDAC and healthy controls.^17^ This panel is composed of specific *N*-glycosylation features, namely glycan branching, sialylation, and antenna-fucosylation and showed an area under the curve (AUC) of 0.81–0.88.^17^ Subsequently, in a longitudinal study by Levink et al,^19^ it was demonstrated that these glycosylation differences occurred 3–50 months before PDAC diagnosis, implying good potential for early detection purposes.

Although promising, this NGT panel has not been evaluated yet in a diverse cohort consisting of individuals with different pancreatic diseases that more closely resembles clinical practice.^17^ Therefore, this study aims to evaluate the reproducibility and generalizability of the NGT panel in discriminating PDAC from a control cohort, consisting of healthy controls and controls with benign pancreatic diseases. Furthermore, this study aims to assess the performance of this NGT panel in combination with CA19-9.

## MATERIALS AND METHODS

### Study Design

This is a case-control study using plasma samples obtained from patients seen at the Department of Gastroenterology and Hepatology at Mayo Clinic Florida. The study was conducted between January 2018 and March 2022. The study was approved by the Institutional Review Board (IRB) of Mayo Clinic Florida under the number 17-005211. All participants have provided written informed consent.

### Study Population

All individuals aged 18 years or older presenting with epigastric symptoms were considered for inclusion in the study. Individuals with neuroendocrine tumors (NET) and all non-PDAC cancer cases (except squamous and basal cell carcinoma in situ) were excluded from the study. The relevant demographics and clinical data were extracted from the medical records.

The case cohort is composed of individuals with newly diagnosed PDAC, confirmed through (cyto-) pathology during EUS or histopathology during surgical resection. Diagnosis was based on the eighth edition of the American Joint Committee on Cancer (AJCC) staging system, with the date of diagnosis defined as the date of malignancy confirmation.^21^

The control group consisted of healthy controls without any pancreatic diseases and those diagnosed with benign pancreatic diseases, such as pancreatitis and (non-)cystic benign pancreatic lesions. Healthy controls were confirmed using imaging [either EUS, abdominal computed tomography (CT) or magnetic resonance imaging (MRI)]. The diagnoses of controls with a benign pancreatic disease were determined using (cyto-) histopathology or imaging. In cases where a more definitive diagnosis was needed, gene panel testing (PancreasSeq) was conducted.^22^ To confirm control status, clinical data from all individuals included were examined up to 24 months after blood collection. Individuals who were diagnosed with PDAC within 24 months after sample collection were reclassified as cases.

### Plasma Sample Collection and Preparation

Venous blood samples were obtained proximate to the date of the imaging. Venous blood was collected from each subject in volumes of 20 mL and placed into 10 mL EDTA Vacutainer tubes. These tubes were then centrifuged at 2000*g* for 10 minutes. The plasma was stored in five 2 mL cryovials, each containing 1 mL of plasma, and frozen at −80 °C until further analysis could be conducted. Of each sample, a 100 µL aliquot was shipped to Leiden University Medical Center (LUMC) on dry ice for measurement of CA19-9 and for analysis of NGTs. The tumor marker CA19-9 was quantified using an Elecsys CA19-9 tumor marker immunoassay based on the monoclonal 1116‑NS‑19‑9 antibody. This tumor marker has a normal reference value of 0.0–37.0 U/mL (99th percentile). The same samples were analyzed according to the *N*-glycomics methodology previously reported by Vreeker et al,^23^ with all samples divided over 3 microtiter plates (MTPs) and subsequently analyzed using the same workflow. Analysts were blinded to the case-control status.

For quality control purposes, each MTP contained 4 replicas of a plasma standard sample (Visucon-F frozen normal control plasma pooled from 20 human donors, Affinity Biologicals) and 6 replicas of a pooled sample constructed from all 245 plasma samples. The *N*-glycomics workflow consisted of an enzymatic release of *N*-glycans from circulatory proteins, linkage-specific sialic acid derivatization, purification, mass spectrometry (MS)-based identification and quantification, and largely automated data extraction and analysis. Briefly, after *N*-glycan release with PNGaseF (Roche Diagnostics), all following pipetting steps were performed in a standardized manner on a Hamilton liquid handling platform. First, sialic acid residues at the nonreducing ends of the complex glycan structures were derivatized into stable end-products, allowing the mass spectrometric differentiation between α2,3-linked and α2,6-linked species. Next, the derivatized glycans were purified using in-house developed cotton-based hydrophilic interaction liquid chromatography (HILIC) micro-tips. The purified glycans were eluted and premixed with super dihydroxybenzoic acid matrix (5 mg/mL in 99% ACN with 1 mmol/L NaOH). The mixture was spotted onto a matrix-assisted laser desorption/ionization (MALDI) AnchorChip target plate (Bruker Daltonics) and measured on a Bruker 15T solariX XR Fourier transform ion cyclotron resonance (FTICR) system. All MS-based glycomics data were curated before further analysis. Replicas from Visucon plasma and a pooled sample were subjected to standard quality check evaluations.

### Statistical Analysis

Baseline characteristics are presented as either the mean with SD or the median with interquartile range (IQR). Logistic regression was performed to calculate odds ratios (ORs) for 6 glycosylation traits (CA2 = di-antennary complex glycans; CA4 = tetra-antennary complex glycans; CFa = antenna-fucosylation of complex glycans; A3F0L = α2,3-sialylated nonfucosylated tri-antennary glycans; A3F0E = α2,6-sialylated nonfucosylated tri-antennary glycans; and A3FE = α2,6-sialylated fucosylated tri-antennary glycans; see Supplementary Table S-1, Supplemental Digital Content 2, http://links.lww.com/MPA/B387 for the formulas of these glycosylation traits) that distinguish PDAC from controls with highest accuracy, based on previous studies.^17,19^ Bonferroni correction was applied to adjust for multiple testing. Boxplots were generated for these NGTs and CA19-9 to visualize the data points. Notably, although literature suggests an association of CFa with age and sex, our study observed only weak or very weak correlations, and therefore we did not adjust for age and sex (Supplementary Tables S-2 and S-3, Supplemental Digital Content 1, http://links.lww.com/MPA/B386).^16^

To determine the diagnostic performance of the NGT panel from Vreeker et al,^17^ the CA19-9 marker, and the combination of these for discriminating PDAC cases from controls (including both healthy controls and those with benign pancreatic diseases), the receiver operating characteristic (ROC) curve and corresponding AUC were calculated. The NGT panel consists of a combination of 3 biologically distinct glycosylation features, namely CA4, A3F0L, and CFa. These 3 were selected based on their inclusion among the top 6 glycosylation traits identified in the previous study as most effective in distinguishing PDAC from non-PDAC, as well as their additional criterion of biological distinctiveness from one another.^17,19^ The sensitivity and specificity were determined from the ROC curve at the point of maximum Youden’s index. The Delong test was used to calculate the 95% CIs for the AUC.^24^ For sensitivity and specificity, the 95% CIs were calculated using the bootstrap method.^25^ In addition, ROC curves were determined for early-stage (I + II) and late-stage (III + IV) PDAC. To determine the diagnostic performance of the markers in discriminating PDAC cases from healthy controls only, the same analyses were performed, focusing on the NGT panel alone, the CA19-9 marker alone, and the combination of the 2.

Lastly, as part of a sensitivity analysis to understand the effect of NETs, the AUC was determined for the above-mentioned NGT panel and CA19-9 in differentiating PDAC from controls, including individuals diagnosed with NET. All analyses were performed using RStudio 2023.12.0.

## RESULTS

**FIGURE 1:**
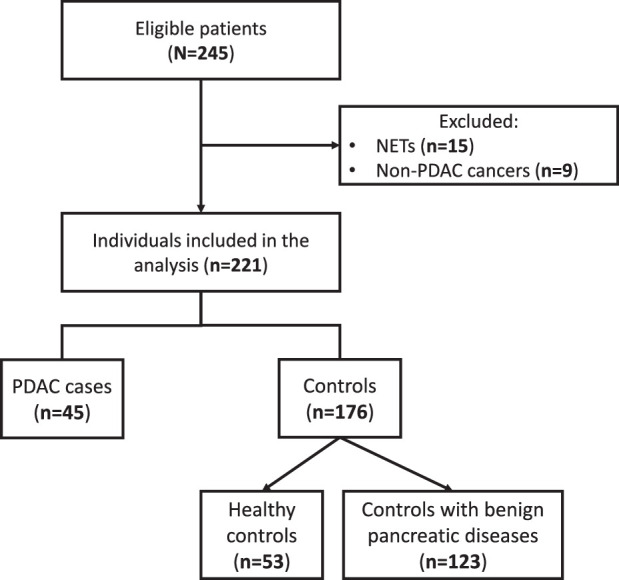
Flowchart depicting the inclusions and exclusions in the study. NET indicates neuroendocrine tumor; PDAC, pancreatic ductal adenocarcinoma.

In total, 221 individuals were included, of whom 45 (20.4%) were diagnosed with PDAC, and 176 (79.6%) served as controls (see Fig. 1 for the flowchart). Of the PDAC group, 11 (24.4%) had stage I, 9 (20.0%) stage II, 6 (13.3%) stage III, and 19 (42.2%) stage IV. A comprehensive description of all PDAC cases can be found in Supplementary Table S-4, Supplemental Digital Content 1, http://links.lww.com/MPA/B386. Among controls, 53 (30.1%) were healthy controls and 123 (69.9%) were controls with a benign pancreatic disease, including intraductal papillary mucinous neoplasms (IPMNs; n=46), non-IPMN cystic lesions (n=18), pancreatitis (n=18), and other benign pancreatic diseases (n=41). The median age of PDAC cases and controls was 72.7 (IQR: 14.7) and 65.6 years (IQR: 18.1), respectively. Among patients diagnosed with PDAC, 24 cases (53.3%) were male versus 78 cases (44.3%) in the control group. The BMI in PDAC cases and controls was similar [26.4 (IQR 7.1) and 26.6 (IQR 7.7)], respectively. A detailed description of the study population is provided in Table 1.

**TABLE 1:** Baseline Characteristics Categorized Into Cases and Controls

	PDAC (n=45)	Controls (n=176)	Healthy controls only (n=53)
Age, median (IQR)	72.7 (14.7)	65.6 (18.1)	54.1 (21.5)
Sex, male, n (%)	24 (53.3)	78 (44.3)	18 (34.0)
BMI, median (IQR)	26.4 (7.1)	26.6 (7.7)	26.6 (6.5)
Follow up, median (IQR)	54.1 (17.9)	57.0 (21.1)	59.0 (30.1)
Smoking, n (%)			
Current smoker	4 (9.1)	19 (10.8)	10 (18.9)
Previous smoker	19 (43.2)	57 (32.4)	12 (22.6)
Never smoked	19 (43.2)	96 (54.5)	30 (56.6)
Unknown	2 (4.5)	4 (2.3)	1 (1.9)
Alcohol use, n (%)			
>3 times a week	9 (20.0)	23 (13.1)	7 (13.2)
<3 times a week	8 (17.8)	53 (30.1)	16 (30.2)
Currently not	6 (13.3)	24 (13.6)	7 (13.2)
No	14 (31.1)	57 (32.4)	16 (30.2)
Unknown	8 (17.8)	19 (10.8)	7 (13.2)
Diabetes, n (%)			
Present	15 (33.3)	30 (17.0)	4 (7.5)
Not present	30 (66.7)	144 (82.3)	49 (92.5)
Unknown	0 (0.0)	1 (0.6)	0 (0.0)
History of acute pancreatitis, n (%)	3 (6.7)	47 (27.0)	13 (25.5)
History of chronic pancreatitis, n (%)	0 (0.0)	17 (9.9)	0 (0.0)
History of pancreatic cancer, n (%)	1 (2.2)	2 (1.1)	0 (0.0)
Family history of pancreatic cancer, n (%)	6 (13.6)	28 (16.3)	6 (11.3)
FNA performed, n (%)	41 (91.1)	62 (36.5)	1 (2.0)
Classification, n (%)			
Controls	0 (0.0)	53 (30.1)	53 (100.0)
IPMN	0 (0.0)	46 (26.1)	0 (0.0)
Non-IPMN cystic lesions	0 (0.0)	18 (10.2)	0 (0.0)
Pancreatitis	0 (0.0)	18 (10.2)	0 (0.0)
PDAC	45 (100.0)	0 (0.0)	0 (0.0)
Other	0 (0.0)	41 (23.3)	0 (0.0)
AJCC stage, n (%)			
Stage I	11 (24.4)	NA	NA
Stage II	9 (20.0)	NA	NA
Stage III	6 (13.3)	NA	NA
Stage IV	19 (42.2)	NA	NA

Other consisted of acellular/inadequate (n=17), atypical (n=1), fatty pancreas (n=6), and unspecified benign disease (n=16).

AJCC indicates American Joint Committee on Cancer; BMI, body mass index; CVD, cardiovascular diseases; FNA, fine-needle aspiration; IPMN, intraductal papillary mucinous neoplasm; IQR, interquartile range; n, number; NA, not applicable; PDAC, pancreatic ductal adenocarcinoma.

### Association Between the 6 *N*-Glycosylation Traits, CA19-9, and Pancreatic Ductal Adenocarcinoma

**FIGURE 2:**
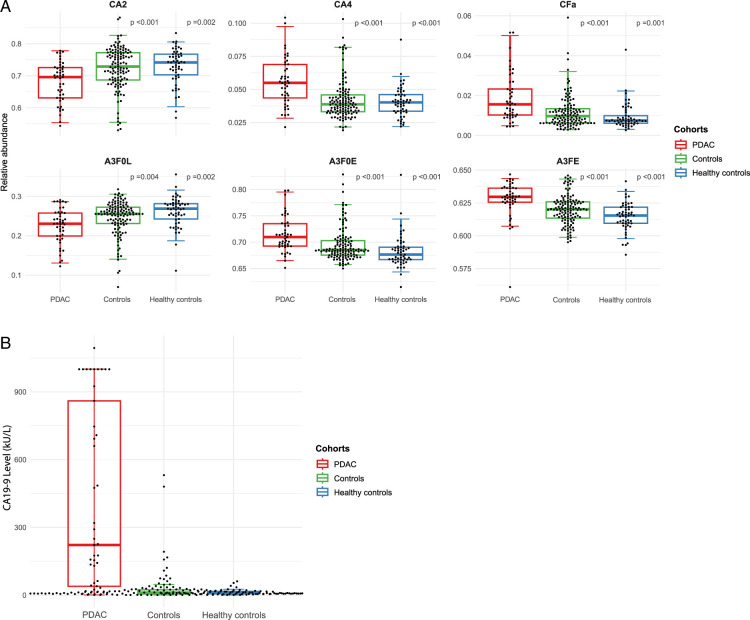
(A) Boxplots with 95% CIs showing the relative abundance of the *N*-glycosylation traits (NGTs) in 3 cohorts: individuals with pancreatic ductal adenocarcinoma (red), the control cohort, consisting of healthy controls and controls with benign pancreatic diseases (green), and healthy controls only (blue). All observed trends align with those previously reported (Vreeker et al)^17^ (B) Boxplots with 95% CIs showing the CA19-9 level (kU/L) in 3 cohorts: individuals with pancreatic ductal adenocarcinoma (red), the control cohort, consisting of healthy controls and controls with benign pancreatic diseases (green), and healthy controls only (blue). A3F0E, α2,6-sialylated nonfucosylated tri-antennary glycans; A3F0L = α2,3-sialylated nonfucosylated tri-antennary glycans; A3FE = α2,6-sialylated fucosylated tri-antennary glycans; CA2 indicates di-antennary complex glycans; CA4, tetra-antennary complex glycans; CFa, antenna-fucosylation of complex glycans.

Figure 2A presents boxplots with 95% CIs, illustrating the relative abundance levels of 6 NGTs among individuals with PDAC and controls. Figure 2B shows boxplots with 95% CIs for the levels of CA19-9 in PDAC and controls. The ORs of 6 NGTs in distinguishing PDAC from the controls are presented in Table 2. All 6 NGTs were statistically significant. The ORs for 3 biologically distinct NGTs (CA4, A3F0L, and CFa) in differentiating cases from the total control cohort were 1.68 (95% CI, 1.37–2.10), 0.88 (95% CI, 0.82–0.95), and 2.15 (95% CI, 1.54–3.12), respectively. In comparison to the healthy controls only, the ORs for the cases were 2.14 (95% CI, 1.52–3.22), 0.80 (95% CI, 0.70–0.89), and 3.66 (95% CI, 1.98–7.88), respectively.

**TABLE 2:** Odds Ratios for 6 Glycosylation Traits Distinguishing Pancreatic Ductal Adenocarcinoma Cases From a Control Cohort, Including Healthy Controls and Controls With Benign Pancreatic Diseases

	PDAC compared with controls		PDAC compared with healthy controls only	
Variables	Odds ratio (95% CI)	*P*	Odds ratio (95% CI)	*P*
CA2	0.90 (0.86–0.95)	<0.001	0.86 (0.79–0.93)	0.002
CA4	1.68 (1.37–2.10)	<0.001	2.14 (1.52–3.22)	<0.001
CFa	2.15 (1.54–3.12)	<0.001	3.66 (1.98–7.88)	0.001
A3F0L	0.88 (0.82–0.95)	0.004	0.80 (0.70–0.89)	0.002
A3F0E	1.20 (1.10–1.33)	<0.001	1.42 (1.21–1.72)	<0.001
A3FE	2.08 (1.52–2.94)	<0.001	2.68 (1.76–4.41)	<0.001

Analysis was also performed for healthy controls only. The Bonferroni correction was used to account for multiple testing.

PDAC indicates pancreatic ductal adenocarcinoma.

### The Clinical Performance of the NGT Panel (CA4, A3F0L, and CFa) and CA19-9

**FIGURE 3:**
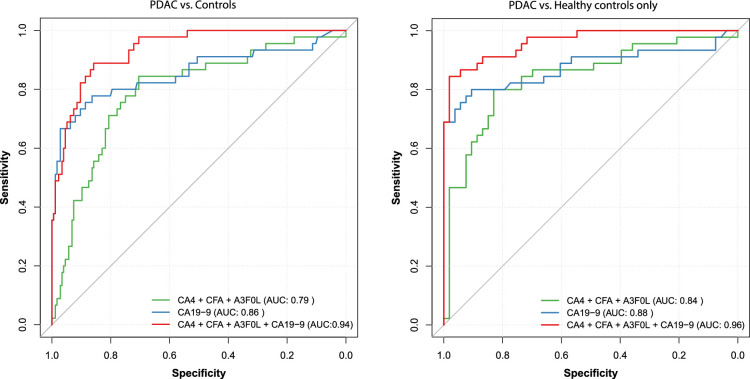
The receiver operating characteristic (ROC) curves illustrate the diagnostic efficacy of the *N*-glycosylation traits panel (CA4, A3F0L, and CFa), CA19-9, and a combination of the 2 in distinguishing pancreatic ductal adenocarcinoma (PDAC) from the controls (healthy controls and controls with benign pancreatic diseases) and from the healthy controls only.

The AUC for the NGT panel (CA4, A3F0L, and CFa) that distinguishes between PDAC cases and the controls was 0.79 (95% CI, 0.72–0.87), with a sensitivity of 0.84 (95% CI, 0.73–0.93) and specificity of 0.70 (95% CI, 0.64–0.77). For CA19-9, the AUC was 0.86 (95% CI, 0.78–0.93) with a sensitivity of 0.76 (95% CI, 0.62–0.87) and specificity of 0.89 (95% CI, 0.84–0.93). When combining the NGT panel with CA19-9, the AUC increased to 0.94 (95% CI, 0.90–0.97) with a sensitivity of 0.89 (95% CI, 0.78–0.98) and specificity of 0.86 (95% CI, 0.81–0.91). See Figure 3 for the ROC curves. The complete list of cutoff values with corresponding sensitivities and specificities for all 3 models is provided in Supplementary Table S-5, Supplemental Digital Content 3, http://links.lww.com/MPA/B388.

The AUC value for the NGT panel in differentiating PDAC cases from healthy controls only was 0.84 (95% CI, 0.76–0.92), with a sensitivity of 0.80 (95% CI, 0.67–0.91) and specificity of 0.83 (95% CI, 0.74–0.92). For CA19-9, the AUC was 0.88 (0.80–0.96) with a sensitivity of 0.80 (95% CI, 0.67–0.91) and a specificity of 0.91 (95% CI, 0.81–0.98). When combining the NGT panel with CA19-9, the AUC increased to 0.96 (95% CI, 0.93–0.99), with a sensitivity of 0.84 (95% CI, 0.73–0.93) and specificity of 0.98 (95% CI, 0.94–1.00).

As a sensitivity analysis, an ROC curve was created for the purpose of distinguishing PDAC from controls, including individuals with a NET diagnosis (n=15). This resulted in an AUC for the NGT panel in combination with CA19-9 of 0.94 (95% CI, 0.90–0.97) with a sensitivity of 0.84 (95% CI, 0.73–0.93) and specificity of 0.88 (95% CI, 0.83–0.92; Supplementary Figure S-1, Supplemental Digital Content 1, http://links.lww.com/MPA/B386).

### The Clinical Performance of the NGT Panel in Combination With CA19-9 in Early- and Late-stage PDAC

When the NGT panel was assessed in combination with CA19-9 in early-stage (I + II) PDAC, an AUC of 0.91 (95% CI, 0.85–0.96) was observed, accompanied by a sensitivity of 0.95 (95% CI, 0.85–1.00) and specificity of 0.71 (95%CI, 0.64–0.77). In late-stage (III + IV) PDAC, the AUC increased to 0.97 (95% CI, 0.94–0.99) with a sensitivity of 1.00 (95% CI, 1.00–1.00) and specificity of 0.83 (95% CI, 0.77–0.88). See Supplementary Figures S-2A, B, Supplemental Digital Content 1, http://links.lww.com/MPA/B386 for the corresponding plots.

## DISCUSSION

This study demonstrates that the combination of the NGT panel, consisting of antennarity (CA4), sialylation (A3F0L), and fucosylation (CFa), with CA19-9 offers an accurate way to distinguish PDAC from non-PDAC. This study further shows that the NGT panel can distinguish individuals with PDAC in a control cohort that more closely resembles clinical practice, further reinforcing the PDAC-specificity of this panel. The performance was consistent in patients with early- and late-stage PDAC. These findings furthermore suggest that the combination of new molecular markers such as NGTs with CA19-9 provides a clinical tool that can be complementary to imaging readouts in pancreatic cancer surveillance programs.^26^

This research builds upon the work of Vreeker et al^17^ and Levink et al,^19^ in which 6 NGTs were selected that could differentiate PDAC from healthy controls. It was furthermore found that these NGTs appeared 3–50 months before PDAC diagnosis, indicating their potential use as an early detection biomarker.^19^ From these 6 NGTs, 3 biologically distinct NGTs were combined, namely CA4, A3F0L, and CFa, for further performance evaluation.^17,19^ This NGT panel resulted in an AUC of 0.84 (95% CI, 0.76–0.92) in differentiating PDAC cases from healthy controls. This finding was consistent with that of Vreeker et al,^17^ where an AUC of 0.88 was reported in the discovery cohort and an AUC of 0.81 in the internal validation. In addition, we examined the performance of these traits in differentiating PDAC from a control cohort consisting of both healthy controls and controls with benign pancreatic diseases and found an AUC of 0.79 (95% CI, 0.72–0.87). Notably, our present study examined plasma samples, whereas previous studies analyzed the *N*-glycome in serum. Although serum and plasma *N*-glycome exhibit differences, these differences are minor.^27^ In fact, the similarity of our findings with the previous studies further suggests that these differences are negligible.

Moreover, we have also examined the NGTs, consisting of 6 previously selected traits, for differentiating PDAC from non-PDAC.^17,19^ In our study, we observed an upregulated expression of tetra-antennary species of complex glycans (CA4), antenna-fucosylation of complex glycans (CFa), α2,6-sialylation of nonfucosylated tri-antennary glycans (A3F0E), and α2,6-sialylation of fucosylated tri-antennary glycans (A3FE) in individuals with PDAC compared with controls. Conversely, within the same groups, we observed a downregulation of di-antennary species of complex glycans (CA2) and α2,3-sialylation of nonfucosylated tri-antennary glycans (A3F0L). Our findings align with those of the previous studies, demonstrating similar trends in all 6 NGTs.^19^ Remarkably, our previous 2 studies were conducted in Dutch cohorts, whereas this study was carried out in a cohort from the United States, showing geographic generalizability. Similar findings were reported in a study by Nouso et al,^28^ in which the serum *N*-glycome was examined in 92 patients with PDAC and 243 healthy volunteers. The study revealed an increase in tetra-antennary species of complex glycans (CA4) and an increase in fucosylation and sialylation in patients with PDACs compared with healthy volunteers. These findings are further confirmed by McDowell et al,^29^ who investigated the *N*-glycome in pancreatic tumor tissues and compared them to the adjacent healthy tissue, also finding increased sialylation and fucosylation, and suggesting differences at the local tissue level. Another study conducted by Akimoto et al^30^ on IPMNs demonstrated that bi-, tri-, and tetra-antennary complex-type glycans with fucose residues were more prevalent in patients with invasive carcinomas compared with individuals with low-, intermediate-, and high-grade IPMNs. This suggests that premalignant stages of PDAC could be differentiated from those of invasive carcinoma and that NGTs could play a role in future clinical decision-making.

The potential causality and underlying mechanisms that link protein glycosylation and PDAC remain poorly understood.^31^ It is reported that altered glycosylation profiles may influence or may result from processes such as tumor growth, differentiation, transformation, cell adhesion, pathogen recognition, and immune surveillance.^15,16^ For instance, glycosylation has been found to be involved in the process of epithelial-mesenchymal transition in various cancers, an important step in carcinogenesis that promotes increased cell migration.^32^ Another example is the hypothesis that certain glycans can mimic host antigens, allowing cancer cells to evade immune detection and thereby protecting them from immune system attacks.^32^ Further studies are needed to elucidate the precise role of glycosylation and its associated pathways in the development of PDAC.

Distinguishing PDAC from benign pancreatic diseases in clinical practice remains challenging due to overlapping symptoms and imaging results.^33^ For example, early imaging signs of PDAC can resemble those of acute pancreatitis, making them indistinguishable from one another, even though they require different treatment approaches.^34^ This similarity can lead to delayed diagnosis as well as false-positive findings.^6,34^ Providing ways to distinguish between PDAC and benign pancreatic diseases will facilitate early detection and reduce the number of individuals undergoing surgery for benign lesions. Notably, the NGT panel with CA19-9 demonstrated the capacity to differentiate PDAC from a control cohort consisting of both healthy controls and controls with benign pancreatic diseases (AUC 0.94). This suggests that the biomarker has potential for distinguishing between malignant and benign pancreatic processes.

The performance of CA19-9 in distinguishing PDAC from non-PDAC in our study is similar to that reported in the literature.^35^ A meta-analysis of 23 studies involving a mixed cohort of healthy controls and controls with benign pancreatic diseases found an AUC of 0.83 (95% CI, 0.78–0.89) for CA19-9. In our study, the AUC at PDAC diagnosis was 0.86 (95% CI, 0.78–0.93). When considering only healthy controls, CA19-9 demonstrated an AUC of 0.87 (95% CI, 0.78–0.97) for differentiating PDAC from healthy controls at the time of diagnosis or up to 6 months prior.^36^ However, this AUC dropped to 0.70 (95% CI, 0.60–0.81) when measured 6–12 months before diagnosis.^36^ In our study, we found an AUC of 0.88 (95% CI, 0.80–0.96) in differentiating PDAC from healthy controls at diagnosis. Unfortunately, the performance of the biomarker before PDAC diagnosis was not evaluated due to missing longitudinal samples. It is important to note that the utility of CA19-9, a single glycan epitope, is limited by its effectiveness in low- or non-secretors, who account for up to 10%–30% in the general population.^37,38^ In contrast, the NGT panel does not face this limitation, as it captures a broader spectrum of glycans, which may account for its complementary role when used alongside CA19-9 in PDAC diagnosis.^14^ Building on this, along with our previous findings that NGTs alterations can be detected 3–50 months before PDAC diagnosis, it would be interesting to explore the longitudinal value of combining these biomarkers for early detection of PDAC.^19^

Understanding the setting and context is crucial when using biomarkers in clinical practice. In pancreatic cancer, it is essential to distinguish between screening and surveillance. Screening refers to testing the general population and given the low incidence of PDAC, even a test with 99% specificity would yield a high amount of false-positives.^39^ In contrast, surveillance focuses on monitoring individuals at high risk and requires a highly sensitive test to ensure no PDAC cases are missed.^6^ Studies show that annual imaging alone in pancreatic cancer surveillance is insufficient for diagnosing early-stage PDAC, leading to late-stage PDAC, missed, and interval cancer cases.^8,9^ A sensitive add-on test could facilitate early detection, either by itself or by justifying further testing or shortening of surveillance intervals. The NGT panel in combination with CA19-9 might serve as a valuable complement to imaging in pancreatic cancer surveillance programs. It is important to note that the accuracy of this panel is based on single measurements only. The use of multiple measurements in a longitudinal setting could offer a way to monitor the *N*-glycome and CA19-9 changes in the blood, which could lead to a more accurate diagnosis of PDAC.^19^ As a next step, we intend to evaluate the added value of this NGT panel in combination with CA19-9 in a prospective longitudinal study involving a high-risk surveillance cohort of *CDKN2A* pathogenic variant carriers.^8^

One particularly promising prospect in the logistics of the *N*-glycans is the possibility of combining the measurement with a finger prick instead of doing a blood draw. A study conducted by Vreeker et al^40^ demonstrated that the *N*-glycome found in a finger-prick sample was highly similar to the one obtained from a venipuncture. The finger-prick approach will facilitate the implementation of this test on a large scale, as it is less invasive in comparison to the standard blood collection using a venipuncture.^41^ Moreover, it was shown that *N*-glycan profiles remained stable over time at various temperatures, further supporting the practicality of this method.^40^ This stability makes sample collection convenient for individuals who must travel long distances to the clinic or require routine testing over an extended period of time, as would be the case for HRIs undergoing pancreatic cancer surveillance programs.

There are a few limitations of this study. First, as already mentioned, this study was based on single measurements taken around the time of diagnosis, leaving it unclear whether changes in the *N*-glycome could be used for early detection of PDAC. Second, although the subgroup analysis of early- and late-stage PDAC is informative, it is underpowered and, therefore, no robust conclusions can be drawn regarding the performance of our markers in early-stage PDAC compared to late-stage PDAC. Third, it is important to acknowledge that there are other confounding factors that influence the NGT panel, such as genomic variations, different PDAC morphologies, and effects of medications, that have not been thoroughly studied and thus have not been taken into account.^42,43^ Another point to consider is the underlying mechanism of the glycosylation process. The applied *N*-glycome analysis provides a general overview of glycosylation differences originating from various glycoproteins.^14^ These differences give further insight into glycosylation processes that may be related to PDAC pathogenesis.^14^ While these traits provide an overview of the total glycosylation changes, they do not indicate whether specific glycoproteins are upregulated or downregulated.^14^ Therefore, as a next step, it is important to determine which specific glycoproteins are involved in PDAC using glycoproteomic strategies.^14^

In conclusion, our study demonstrates that the 3 biologically distinct NGTs, in combination with CA19-9, effectively discriminate between PDAC and non-PDAC cases in a diverse cohort. This combination of NGTs and CA19-9 holds promise as a future tool in pancreatic cancer surveillance programs.

## Supplementary Material

SUPPLEMENTARY MATERIAL
