# Supplementary material for: Protein N-Glycosylation Traits Combined With CA19-9 Accurately Distinguish Pancreatic Cancer Cases From Healthy Controls and Benign Pancreatic Diseases
Source: Pancreas. 2025 May 23;54(9):e823–30. doi: 10.1097/MPA.0000000000002517 (PMC12419019; doi:10.1097/MPA.0000000000002517)
Supplement: SUPPLEMENTARY MATERIAL [file mpa-54-e823-s001.docx]

**Table S-2**: Assessment of glycosylation trait CFa for normality assumption

| **Shapiro-Wilk normality test** | |
| --- | --- |
| W | P-value |
| 0.78676 | <0.001* |

* Based on this analysis, the null hypothesis is rejected due to the Shapiro-wilk p-value <0.05. Since the normality assumption is not met, spearman’s rho test is conducted instead of linear regression.

**Table S-3**: Evaluation of the correlation between CFa and age, sex and diabetes mellitus status

| **Spearman’s Rho test** | | | |
| --- | --- | --- | --- |
| **Variable** | **Correlation coefficient** | **P-value** | **Correlation*** |
| Age | 0.09 | 0.194 | Very weak |
| Sex | 0.26 | <0.001 | Weak |
| Diabetes mellitus | -0.20 | 0.002 | Weak |

*Based on the Spearman’s Rho test, only weak or very weak correlations have been found between the CFa glycosylation trait and the variables Age, Sex, and diabetes mellitus.

**Table S-4**. Details of individuals who were diagnosed with pancreatic ductal adenocarcinoma and their corresponding predictions based on the biologically distinct *N*-glycosylation panel.

| No. | Age at diagnosis | Sex | Interval from sample collection to PDAC, months* | Stage | Lesion  Location | Surgery performed |
| --- | --- | --- | --- | --- | --- | --- |
| 1 | 65 | Female | 0 | Stage III | tail | Yes |
| 2 | 84 | Female | 3 | Stage IA | head | Yes |
| 3 | 61 | Female | 5 | Stage III | body | No |
| 4 | 69 | Female | 0 | Stage IIB | head | Yes |
| 5 | 70 | Female | 0 | Stage IV | tail | No |
| 6 | 91 | Male | 1 | Stage IV | head | No |
| 7 | 90 | Male | 0 | Stage IB | head | Yes |
| 8 | 89 | Male | 0 | Stage IIB | head | Yes |
| 9 | 81 | Female | 0 | Stage IA | head | Yes |
| 10 | 79 | Male | 0 | Stage IV | tail | No |
| 11 | 62 | Male | 3 | Stage IB | head | Yes |
| 12 | 66 | Male | 0 | Stage IV | body | No |
| 13 | 79 | Female | 0 | Stage IV | body | No |
| 14 | 85 | Female | 0 | Stage IIB | body | Yes |
| 15 | 66 | Female | 1 | Stage IB | head | Yes |
| 16 | 73 | Female | 0 | Stage IV | head | No |
| 17 | 81 | Female | 1 | Stage IA | body | Yes |
| 18 | 50 | Female | 0 | Stage IV | tail | No |
| 19 | 57 | Female | 0 | Stage IA | head | No |
| 20 | 74 | Female | 0 | Stage IIA | head | No |
| 21 | 63 | Male | 2 | Stage IIA | neck | Yes |
| 22 | 80 | Male | 1 | Stage IIB | head | Yes |
| 23 | 61 | Male | 0 | Stage IB | head | No |
| 24 | 48 | Male | 1 | Stage IB | body | Yes |
| 25 | 76 | Male | 0 | Stage IV | head | No |
| 26 | 78 | Male | 0 | Stage IIB | head | No |
| 27 | 49 | Male | 1 | Stage IV | head | No |
| 28 | 68 | Male | -15 | Stage IV | head | No |
| 29 | 67 | Male | 4 | Stage IV | neck | No |
| 30 | 77 | Female | 0 | Stage III | neck | No |
| 31 | 80 | Female | 0 | Stage IIA | head | No |
| 32 | 71 | Male | 8 | Stage IIB | head | Yes |
| 33 | 76 | Male | 0 | Stage IV | tail | No |
| 34 | 65 | Male | 0 | Stage III | head | No |
| 35 | 84 | Male | 0 | Stage IV | tail | No |
| 36 | 73 | Male | 0 | Stage IV | body | No |
| 37 | 83 | Female | 10 | Stage IB | head | Yes |
| 38 | 58 | Male | 0 | Stage IV | head | No |
| 39 | 81 | Female | 0 | Stage IV | body | No |
| 40 | 69 | Male | 0 | Stage III | body | No |
| 41 | 70 | Male | 0.0 | Stage IV | head | No |
| 42 | 70 | Female | 0.2 | Stage III | head | No |
| 43 | 74 | Female | 3.6 | Stage IB | head | Yes |
| 44 | 90 | Female | -0.6 | Stage IV | head | No |
| 45 | 65 | Male | 0.7 | Stage IV | head | No |

* The interval between plasma sample collection and PDAC diagnosis; a negative value indicates that the sample was collected after PDAC diagnosis.

Abbreviations: NO.=number, PDAC= pancreatic ductal adenocarcinoma.


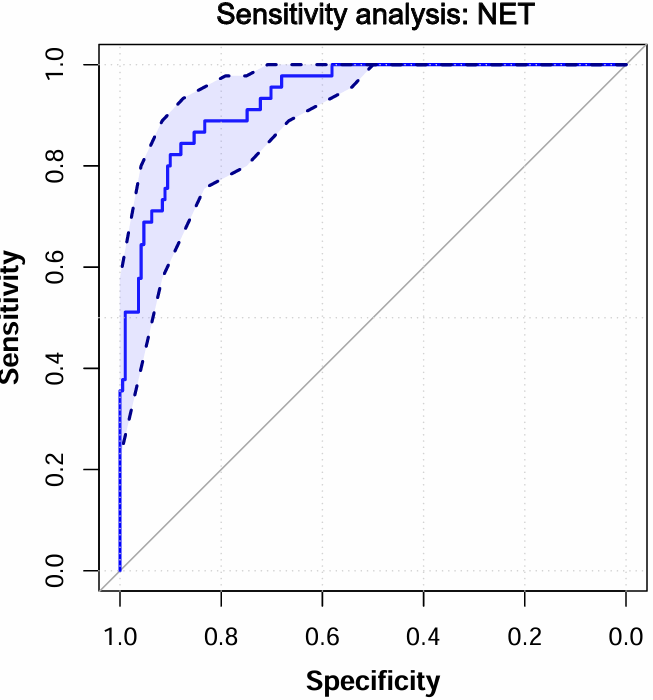


**Figure S-1**: Illustrates the receiver operating characteristic (ROC) curve for the discrimination between pancreatic ductal adenocarcinoma and the control cohort, including individuals with neuroendocrine tumors (n=15).

**
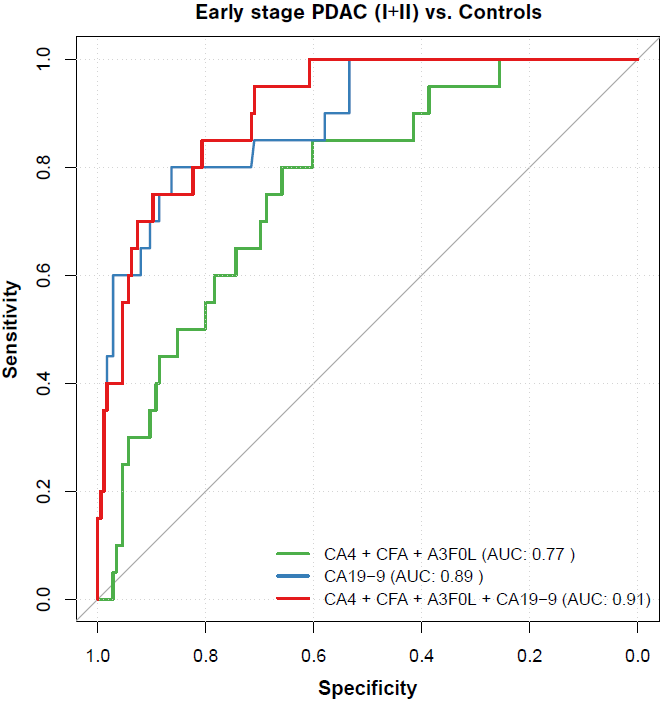
**

**Figure S-2a**: The ROC curves, accompanied by 95% confidence intervals (CIs), illustrate the diagnostic efficacy of the panel of three N-glycosylation traits (CA4, A3F0L and CFa), CA19-9 and the combination of two in distinguishing early-stage (I + II; n= 20) PDAC from controls

**
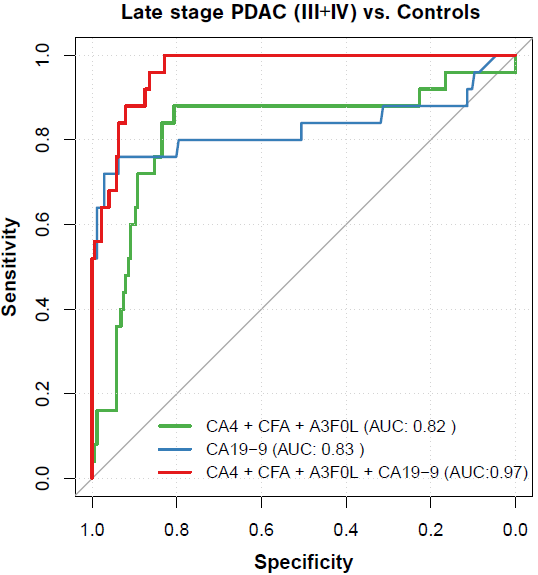
**

**Figure S-2b**: The ROC curves, accompanied by 95% confidence intervals (CIs), illustrate the diagnostic efficacy of the panel of three N-glycosylation traits (CA4, A3F0L and CFa), CA19-9 and the combination of two in distinguishing late-stage (III + IV; n= 25) PDAC from controls
